# Association of Klotho gene polymorphism with hypertension and coronary artery disease in an Iranian population

**DOI:** 10.1186/s12872-018-0971-5

**Published:** 2018-12-14

**Authors:** Hamed Akbari, Gholamreza Asadikaram, Hamid Aria, Saba Fooladi, Sina Vakili, Mohammad Masoumi

**Affiliations:** 1 Neuroscience Research Center, Institute of Neuropharmacology, Kerman University of Medical Sciences, Kerman, Iran; 2 Endocrinology and Metabolism Research Center, Institute of Basic and Clinical Physiology Sciences, Kerman University of Medical Sciences, Kerman, Iran; 3 Department of Biochemistry, School of Medicine, Kerman University of Medical Sciences, Kerman, Iran; 4 Department of Immunology, School of Medicine, Shiraz University of Medical Sciences, Shiraz, Iran; 5 Bioinformatics and Computational Biology Research Center, Shiraz University of Medical Sciences, Shiraz, Iran; 6 Student Research Committee, School of Medicine, Kerman University of Medical Sciences, Kerman, Iran; 7 Biochemistry Department, School of Medicine, Shiraz University of Medical Sciences, Shiraz, Iran; 8 Cardiovascular Research Center, Institute of Basic and Clinical Physiology Sciences, Kerman University of Medical Sciences, Kerman, Iran

**Keywords:** Coronary artery disease, Hypertension, Klotho, Polymorphism

## Abstract

**Background:**

Klotho, possibly an age-regulating protein, is considered an important factor contributing to the lifespan and pathophysiology of hypertension and coronary artery disease (CAD). The present study was carried out aiming to investigate the association of Klotho-rs564481 (C1818T) gene polymorphism with hypertension and CAD.

**Methods:**

A total of 286 CAD-suspicious subjects were entered into this case-control study. The polymorphism was investigated in hypertensive patients with no CAD (H-Tens, *n* = 60); hypertensive patients with CAD (CAD + H-Tens, *n* = 95); CAD patients with no hypertension (CAD, *n* = 61); and non-hypertensive non-CAD subjects, which were regarded as the control group (Ctrl, *n* = 70). Genotype and allele frequencies were assessed using polymerase chain reaction-restriction fragment length polymorphism (PCR-RFLP) method.

**Results:**

A significant difference was found in allele frequency of Klotho C1818T among the four research groups (*P* = 0.03). It was also found that wild-type homozygote subjects were negatively associated with hypertension as compared to heterozygote ones (OR = 0.07 [95% CI: 0.008–0.69] *P* = 0.02). Moreover, in the subgroups older than 57 years old, dominant genetic model demonstrated a negative association with CAD combined with hypertension (OR = 0.31 [95% CI: 0.10–0.95] *P* = 0.04).

**Conclusions:**

In conclusion, Klotho C1818T variant may be associated with a decreased risk of hypertension. Moreover, aging enhanced positive effects of the Klotho polymorphism on CAD combined with hypertension, indicating the possibility that the KLOTHO gene might play a part in the age-related occurrence of CAD combined with hypertension.

## Background

Coronary artery disease (CAD) is characterized as a chronic inflammatory disease and represents one of the main causes of worldwide morbidity and mortality [1]. An impressive body of evidence has proven the role of hypertension in development of CAD [2]. CAD and hypertension are believed to be multifactorial diseases [3–7]. Furthermore, ample evidence has revealed that the metabolic, genetic, and environmental factors are also involved in susceptibility to CAD and hypertension [8, 9]. Recent advances in disease biology and understanding of their molecular mechanisms have led to reporting various susceptible genes involved in incidence of these diseases. In this regard, identification of these genes along with environmental factors is essentially important [10, 11].

Klotho has been recently evaluated in atherosclerosis and is assumed to be an age-regulating protein [12, 13]. This gene is located on human chromosome 13 (13q12), spanning about 50 kb in length and consisting of 5 exons and 4 introns [14]. Genetic studies have shown that Klotho gene variants are associated with atherosclerotic disease [15, 16], and only one study reported a significant correlation between Klotho plasma levels and a reduction in the risk of cardiovascular disease (CVD) [17]. Previous studies showed a complex syndrome in Klotho-deficient mice in which two remarkable changes are arteriosclerosis and endothelial dysfunction [18, 19]. Furthermore, it has been reported that adenovirus-mediated Klotho gene delivery increased nitric oxide (NO) synthesis, improved vascular endothelial dysfunction, and decreased blood pressure (BP) [20]. Therefore, based on the previous findings, Klotho gene is said to contribute to regulation of endothelial function through pathways mediated by NO.

So far, over 10 single nucleotide polymorphisms (SNPs) have been reported in human Klotho gene [12], and a growing body of evidence has shown the relationship between Klotho SNPs, susceptibility to CAD and endothelial dysfunction. [16]. Recently, it has been demonstrated that Klotho C1818T (rs564481) polymorphism, located in the exon 4 of the human Klotho gene, is associated with the risk of CAD, bone mineral density, systolic BP, and vascular dysfunction [12, 20, 21]. Therefore, Klotho gene may be involved in the pathophysiology of CAD. In spite of genetic-related studies to identify the links of Klotho SNPs with changes in related disease risk, the exact mechanisms have not yet been fully elucidated. Since endothelial dysfunction is the one of the etiological factors of CAD and hypertension, it was hypothesized that the polymorphism of the human Klotho gene is involved in CAD and hypertension.

In this content, the objective of this study was to investigate the possible association of the Klotho gene polymorphism with hypertension and CAD.

## Methods

### Subjects

Two hundred eighty-six CAD-suspected patients, who were diagnosed as candidates of coronary angiography, were included. All participants were selected from Shafa Hospital of Kerman University of Medical Sciences, Kerman, Iran (from July 2014 to December 2015). Subjects’ selection process and methodology were according to our previous studies [22–24]. Briefly, all CAD-suspected participants were engaged in selective coronary angiography [25] as they were symptomatic, had a history of hospitalization in CCU, or showed evidence of myocardial ischemia in noninvasive investigations such as exercise test or perfusion imaging. Additionally, CAD was defined as luminal diameter with 50% or more narrowing in the main coronary artery vessels whereas those without any plaque were diagnosed as normal (control group). Moreover, hypertension was defined as BP > 140/90 mmHg or medication use (beta blockers, angiotensin-converting enzyme inhibitor (ACEI), angiotensin receptor blocker (ARB), calcium channel blocker (CCB), and diuretics) for reducing high blood pressure. The subjects discontent with participation in the study were excluded, moreover, patients with tumors, respiratory diseases, congenital heart disease, cerebral infarction, diabetes or chronic kidney diseases, and autoimmune diseases were excluded as well. Subjects were then divided into four groups [22–24]: 1) hypertensive with no CAD (H-Tens, *n* = 60); 2) CAD with hypertension (CAD + H-Tens, *n* = 95); 3) CAD with no hypertension (CAD, *n* = 61); and 4) non-hypertensive non-CAD as the control group (Ctrl, *n* = 70). This study conforms to the *Declaration of Helsinki* regarding research involving human subjects and is approved by the ethics committee of Kerman University of Medical Sciences, Kerman, Iran (IR.KMU.REC/93/286). All participants signed the written informed consents form.

### Samples and data collection

Demographic data was gathered via questionnaire. Body mass index (BMI) was calculated as weight divided by square of height (kg/m2). A 10-ml blood sample was drawn and poured into vacutainer tubes containing EDTA. The fasting blood sugar (FBS), triglycerides (TG), total cholesterol (TC), high-density lipoprotein cholesterol (HDL-c), low-density lipoprotein cholesterol (LDL-c), sodium (Na), potassium (K), creatinine (Cr), and urea were measured using standard kits (Pars Azmoon, Iran). Blood pressure was taken from the right arm in the sitting position (with 10 min-intervals) using an automatic sphygmomanometer, and the average of two readings was used. Systolic blood pressure ≥ 140 mmHg and diastolic blood pressure ≥ 90 mmHg and/or receiving antihypertensive medication were the diagnostic criteria of hypertension [22–24].

### Genotyping of klotho polymorphism

Genomic DNA was extracted from whole blood samples using “salting-out” method with slight modifications [26]. Isolated genomic DNA was stored at − 20 °C until the polymerase chain reaction (PCR). The Klotho C1818T gene polymorphism was analyzed by the polymerase chain reaction-restriction fragment length polymorphism (PCR-RFLP) method as described below. Primers used for PCR were: 5’-TGTCTCAGTTTACCGACCTGAATGT-’ (forward), 5’-ATTCATCGTTATCCAAAGCTTGACG-3′ (reverse). The PCR amplification was done using a Corbett Research thermocycler (Qiagen Company, Germany). PCR conditions were as follows: initial denaturation at 95 °C for 5 min followed by 31 cycles of amplification including denaturation (at 95 °C, 30 s), annealing (at 66 °C, 30 s), extension (at 72 °C, 30 s), and the final extension (at 72 °C 5 min). A 10 μl of. PCR products were digested with *Mph1103I* restriction endonucleases (Thermo Scientific, USA) at 37 °C for approximately 12 h overnight. The PCR fragments were electrophoresed on 1.5% (*w*/*v*) agarose gel and visualized by ethidium bromide staining. Finally, three genotypes of Klotho C1818T were as follows: CC (452 bps), CT (272, 180, and 452 bps), and TT (272, 180 bps).

### Statistical analysis

All continuous variables were expressed as mean ± standard deviation (M ± SD) and categorical variables were presented as numbers (percentages). The Kolmogorov–Smirnov test was used to determine the normality of data. The differences between groups (more than two groups) were analyzed using one-way analysis of variance (ANOVA)/Kruskal-Wallis with post-hoc Tukey/Mann-Whitney U tests as well as Chi-square/Fisher’s exact tests. Hardy–Weinberg equilibrium (HWE) for Klotho polymorphism was performed by the Chi-square test. Multinomial logistic regression was also performed to evaluate the independent roles of these genotypes against hypertension and CAD risk. The statistical analyses were performed using SPSS software version 23.0 for Windows (IBM/SPSS Inc., New York, USA). Statistical significance was assumed at *P*-values < 0.05.

## Results

### Demographics analysis

Comparison of participants’ demographic and clinical characteristics among the four study groups are presented in Table 1. It was reported that there are significant differences in the four groups in terms of age and sex (*P* = 0.003 and *P* = 0.001, respectively). In addition, it was also revealed that BMI and Cr were statistically significant among the four groups (*P* = 0.016 and *P<*0.001, respectively). However, no significant difference was witnessed in FBS, TG, TC, HDL-c, LDL-c, Na, K, and Urea between four study groups (*P* > 0.05 for all comparisons).

**Table 1:** Comparison of demographics and biochemical parameters of subjects among the four groups

Variables	H-Tens (*N* = 60)	CAD + H-Tens (*N* = 95)	CAD (*N* = 61)	Ctrl (*N* = 70)	*P*-value
Age, years	54.5 ± 9.4	58.2 ± 9.5	57.2 ± 9.6	52.9 ± 11.1	0.003*
Sex (m/f)	23/37	59/36	44/17	33/37	0.001*
BMI (kg/m^2^)	26.9 ± 5.3	24.6 ± 4.5	24.1 ± 4.2	25.1 ± 4.7	0.016*
FBS (mg/dl)	95.1 ± 12.0	91.9 ± 10.2	92.2 ± 10.5	94.6 ± 12.6	0.182
TG (mg/dl)	118.9 ± 59.2	114.6 ± 70.8	109.1 ± 47.0	104. ± 36.0	0.840
TC (mg/dl)	142. ± 32.4	138.1 ± 39.2	145.0 ± 42.6	140.9 ± 41.5	0.634
HDL-c (mg/dl)	38.3 ± 9.3	37.4 ± 11.3	38.9 ± 12.1	40.7 ± 10.0	0.276
LDL-c (mg/dl)	78.6 ± 30.9	77.6 ± 33.2	84.2 ± 35.4	78.3 ± 37.4	0.656
Na (mmol/l)	139.4 ± 3.1	139.3 ± 3.0	140.3 ± 2.6	138.9 ± 3.0	0.143
K (mmol/l)	4.3 ± 0.4	4.2 ± 0.4	4.4 ± 0.4	4.3 ± 0.4	0.101
Cr (mg/dl)	0.9 ± 0.2	1.0 ± 0.2	1.1 ± 0.1	1.0 ± 0.2	<0.001*
Urea (mg/dl)	34.9 ± 9.8	36.8 ± 11.7	36.0 ± 10.3	34.2 ± 12.7	0.514

H-Tens = Hypertension; CAD + H-Tens = Coronary artery disease and hypertension; CAD = Coronary artery disease; Ctrl = Control. Values are presented as mean ± SD. The differences of continuous variables among the study groups were analyzed using one-way analysis of variance (ANOVA)/Kruskal-Wallis with post-hoc Tukey/Mann-Whitney U tests. *Significant difference (*P*<0.05)

### Genotype and allele frequencies distribution of Klotho C1818T among four groups

Genotype and allele frequencies distribution of Klotho C1818T gene polymorphism was compared among the four study groups, the details of which are listed in Table 2. A significant difference was found in allele frequency of Klotho C1818T between the four groups (*P* = 0.03). However, no such a difference was reported regarding the genotype frequency (*P* = 0.13). The C allele of Klotho C1818T gene polymorphism was found at 64, 75, 61 and 68% frequencies and the T allele was observed at 36, 25, 39 and 32% frequencies in H-Tens, CAD + H-Tens, CAD, and control groups, respectively (Table 2). The TT genotype of Klotho C1818T gene polymorphism was found at nearly low frequencies of 10, 5, 13 and 11% in H-Tens, CAD + H-Tens, CAD, and control groups, respectively. The genotype and allele frequencies conform to the HWE (Table 2).

**Table 2:** Comparison of genotype and allele frequency of Klotho C1818T among the four groups

Groups SNPs		(1) H-Tens (*N* = 60)	(2) CAD + H-Tens (*N* = 95)	(3) CAD (*N* = 61)	(4) Ctrl (*N* = 70)	*P*-value			
						1 vs. 4	2 vs. 4	3 vs. 4	*1&2&3&4*
C1818T (rs564481) Total Subjects									
CC		23 (38%)	53 (56%)	21 (34%)	33 (47%)	0.50	0.27	0.33	0.13
CT		31 (52%)	37 (39%)	32 (52%)	29 (41%)				
TT		6 (10%)	5 (5%)	8 (13%)	8 (11%)				
Allele C		77 (64%)	143 (75%)	74 (61%)	95 (68%)	0.45	0.14	0.22	0.03*
Allele T		43 (36%)	47 (25%)	48 (39%)	45 (32%)				
HWE	X^2^	0.92	0.2	0.6	0.18				
	*P*-value	0.41	0.79	0.59	0.78				
C1818T (rs564481) Age < 57 years									
CC		14 (41%)	26 (62%)	11 (42%)	25 (58%)	0.28	0.39	0.42	0.28
CT		17 (50%)	15 (36%)	11 (42%)	14 (33%)				
TT		3 (9%)	2 (2%)	4 (15%)	4 (9%)				
Allele C		45 (66%)	67 (80%)	33 (63%)	64 (74%)	0.30	0.39	0.19	0.10
Allele T		23 (34%)	17 (20%)	19 (37%)	22 (26%)				
HWE	X^2^	0.46	0.47	0.20	0.90				
	*P*-value	0.71	0.99	0.68	0.42				
C1818T (rs564481) Age ≥ 57 years									
CC		9 (35%)	27 (51%)	10 (29%)	8 (30%)	0.89	0.16	0.90	0.39
CT		14 (54%)	23 (43%)	21 (60%)	15 (56%)				
TT		3 (12%)	3 (6%)	4 (11%)	4 (15%)				
Allele C		32 (62%)	77 (72%)	40 (59%)	31 (57%)	0.81	0.10	0.98	0.22
Allele T		20 (38%)	29 (28%)	28 (41%)	23 (43%)				
HWE	X^2^	0.49	0.02	1.95	0.49				
	*P*-value	0.69	0.89	0.29	0.70				

(1): H-Tens = Hypertension; (2): CAD + H-Tens = Coronary artery disease and hypertension; (3): CAD = Coronary artery disease; (4): Ctrl = Control. Values are presented as number (percentage). Genotypes and allele frequencies were compared among groups using chi-square/Fisher’s exact tests. *Significant difference (*P*<0.05)

Extensive studies have unveiled Klotho’s anti-aging effects as well as differential impacts of age on the association of Klotho polymorphism with CAD [20, 27]. Accordingly, we divided the participants into two subgroups, namely, younger and older than 57 years, on the basis of the median for the age of all the subjects [20]. As regards subjects younger and older than 57 years old, the genotypes and allele frequency of Klotho C1818T gene polymorphism were not reported to be significantly different among the four study groups (*P* > 0.05 for all comparisons). Moreover, as of total subjects as well as those younger and older than 57 years old, the genotypes and allele frequency were not significantly different in two-by-two comparisons between the four study groups (*P* > 0.05 for all comparisons). The genotype and allele frequencies were in compliance with HWE (Table 2).

### Results of multinomial regression models

Results of multinomial regression model, applied for further data analysis by adjusting for age, sex, BMI, and Cr are presented in Table 3, indicating that Klotho C1818T homozygotes type was a significant predictor of hypertension in the all-subjects category. It was found that wild-type homozygote subjects were negatively associated with hypertension as compared to C allele carriers (OR = 0.07 [95% CI: 0.008–0.69] *P* = 0.02). Notably, in subjects younger than 57 years, neither in recessive model nor in dominant model was a significant association observed between the Klotho C1818T variant and the risk of CAD, hypertension, and CAD combined with hypertension. Moreover, in the subgroups older than 57 years old, dominant genetic model demonstrated a negative association with CAD combined with hypertension (OR = 0.31 [95% CI: 0.10–0.95] *P* = 0.04) while no significant association was found in recessive model as well as in both genetic models of hypertensive and CAD groups (Table 3).

**Table 3:** Association Klotho C1818T polymorphic genotypes and the risk of CAD and hypertension with multinomial regression

Model of inheritance		H-Tens vs. Ctrl		CAD + H-Tens vs. Ctrl		CAD vs. Ctrl	
		*P-*value	Adjusted OR [95% CI]	*P-*value	Adjusted OR [95% CI]	*P-*value	Adjusted OR [95% CI]
C1818T							
Total subjects	(CT + CC) vs. (TT)^1^	0.02*	**0.07 [0.008–0.69]**	0.17	0.35 [0.08–1.56]	0.88	1.09 [0.33–3.64]
	(CC) vs. (CT + TT)^2^	0.27	1.52 [0.71–3.27]	0.14	0.59 [0.29–1.19]	0.34	1.46 [0.67–3.20]
Age<57	(CT + CC) vs. (TT)	0.53	0.53 [0.08–3.44]	0.19	0.19 [0.01–2.30]	0.86	1.17 [0.19–7.08]
	(CC) vs. (CT + TT)	0.15	2.13 [0.76–6.00]	0.99	1.00 [0.37–2.67]	0.30	1.82 [0.59–5.59]
Age ≥ 57	(CT + CC) vs. (TT)	0.19	0.56 [0.83–5.32]	0.86	0.83 [0.09–7.34]	0.61	1.66 [0.23–11.66]
	(CC) vs. (CT + TT)	0.64	0.74 [0.21–2.58]	0.04*	**0.31 [0.10–0.95]**	0.92	1.06 [0.32–3.59]

CAD = Coronary artery disease; H-Tens = Hypertension; CAD + H-Ten = Coronary artery disease and hypertension; Ctrl = Control; OR = Odds ratio; 95% CI = Confidence interval. *Significant difference (*P*<0.05)

^1^Recessive model; ^2^Dominant model: Adjusted for age, sex, BMI, and Cr using the multinomial regression model. *Significant difference (*P*<0.05)

## Discussion

Cardiovascular disorders, considered as multifactorial diseases, might be caused by environmental and genetic factors, and bring about high rates of worldwide morbidity and mortality [28]. Currently, little is known about the complex interplay between the genetic factors and CVDs. Previous genetic studies conducted on humans have implied the possible association of Klotho gene polymorphisms with longevity and CVDs. Nonetheless, they only succeeded in yielding a limited number of results. Thus, as the need was strongly felt, this study evaluated the Klotho gene polymorphism in Iranian subjects to reveal its possible association with CAD, hypertension, and CAD combined with hypertension. In the present study, Klotho C1818T gene polymorphism was attributed to the decreased risk of hypertension and CAD combined with hypertension.

The association of Klotho gene polymorphism with hypertension and CAD was assessed. At the rs564481 locus, the Klotho C1818T gene polymorphism was associated with decreased risk of hypertension in all subjects category as well as a decreased risk of association with CAD combined with hypertension in those subjects older than 57 years. However, no significant association was observed in subjects younger than 57 years subgroups. In the previous reports, the frequency of T allele carriers were 0.41 [29], 0.17 [12] and 0.22 [19] in Caucasians, Japanese healthy subjects and ischemic stroke Korean females, respectively, which resembled those of the present study, indicating a lower frequency of T alleles among the Asian population. The difference between Caucasian and the Asian populations was most likely due to the racial-ethnic discrepancies. In the Asian population, the Klotho C1818T gene polymorphism was associated with an increased risk of CADs, hypertension, glucose metabolism, and lipid levels [12, 15, 20, 30]. Results of a study conducted by Rhee et al. showed that subjects with CC genotype had almost three-fold higher risk of CAD compared to those with T allele carriers [20], suggesting the CC genotype as a predictor for CAD in the Korean population. However, the present study showed that T allele carriers decreased the hypertension and/or CAD risk. Moreover, in stark contrast to the present study, a research conducted in China reported the TT genotype of Klotho C1818T as a susceptibility factor for coronary heart disease [31]. In a systematic review and meta-analysis conducted by Zhang et al., the association of five Klotho SNPs with CVDs was assessed [32], revealing that the Klotho C1818T and G-395A SNPs are the risk factors for coronary heart disease (CHD). However, no significant association was reported between the risk of ischemic stroke and Klotho C1818T SNP, as determined within Indian and Korean populations [18, 19]. In spite of the association of Klotho C1818T gene polymorphism and other Klotho polymorphisms with CHD and CADs as above-mentioned [13, 16, 32], the present study revealed an inverse relationship merely in patients with CAD combined with hypertension. Moreover, the results of the present study are not consistent with the previous studies which indicated the association of Klotho gene polymorphisms with increased risk of hypertension [32]. Contrarily, it showed the association of Klotho gene polymorphisms with decreased risk of hypertension. The discrepancies witnessed here might be because of the differences in the genetic constitution and non-genetic or environmental properties through life. The other illustration possibly endorsing genetic heterogeneity, gene-environment and gene-gene interactions in various populations.

The Klotho’s anti-aging effects and differential effects of age in the association of Klotho polymorphism with CVDs have been well established. Hence, in the present study, the subjects were divided into two subgroups, namely, younger and older than 57. According to the study by Rhee et al. [20], the effects of Klotho C1818T SNP on CAD were enhanced in the subjects younger than 60 years. Considering the identical age-cutoff (60-year olds in Rhee et al., study vs. 57-year olds in the present study), it is safe to claim that the results of the current study strikingly contrasted with those of Rhee et al., suggesting the significant association of Klotho C1818T gene polymorphism in patients over 57 years old suffering from CAD combined with hypertension. On the other hand, they reported the CC genotype as a strong predictor for CAD, a finding not coinciding with that of the present study, in which subjects having CAD combined with hypertension after the age of 57 demonstrated a lower prevalence of TT genotype. Therefore, it is safe to assume that Klotho C1818T polymorphism might be associated with the late atherosclerosis. In spite of hypothetical nature of this deduction, present findings along with further researches to be conducted could clarify the exact mechanism of Klotho SNPs in humans. Furthermore, regarding lack of studies on these age subgroup divisions and important effects of Klotho, a peptide hormone with anti-aging activities, it is recommended that further studies be performed on a larger population to explore the possible age effects.

Some limitations can be considered in interpretation of the results yielded by the study. Firstly, the present study’s participants were restricted to the Iranian southeast populations. Hence, the present findings could not be generalizable to other racial/ethnic populations. Secondly, the results were adjusted for each variable to show the reliable statistical power of our association study by using multinomial regression model analysis. Finally, “H-Tens” group selection was one of the important current study limitations as all H-Tens patients were susceptible to coronary angiography. So, selection of hypertensive patients without any critical of coronary vessel stenosis was a tough and demanding task. Hence a relatively small sample size resulted. However, the present study enjoys some strong points as well. The CAD was diagnosed by coronary angiograms while other studies confirmed the CAD only with an electrocardiogram or a thallium scan. Moreover, to the best of the author’s knowledge, this is the first study reporting the association of Klotho gene polymorphisms with the risk of CAD, hypertension, and CAD combined with hypertension.

## Conclusion

In conclusion, the findings of the present case-controlled study revealed the importance of genetic influences of Klotho polymorphism on hypertension and/or CAD susceptibility among the Iranian southeast population. Furthermore, TT genotype carriers of the Klotho C1818T gene polymorphism decreased the hypertension risk, and aging enhanced the positive effects of the Klotho polymorphism on CAD combined with hypertension, improving the odds that Klotho gene might play a part in the age-related incidence of CAD along with hypertension. Nevertheless, larger studies with different ethnic populations are required to uncover the exact role of Klotho gene in the pathogenesis of CAD, hypertension, and CAD combined with hypertension.

## Abbreviations

ACEI: Angiotensin-converting enzyme inhibitor
ANOVA: Analysis of variance
ARB: Angiotensin receptor blocker
BMI: Body mass index
BP: Blood pressure
CAD + H-Tens: CAD patients with hypertension
CAD: Coronary artery disease
CCB: Calcium channel blocker
CHD: Coronary heart disease
CI: Confidence interval
Cr: Creatinine
Ctrl: Control
CVDs: Cardiovascular diseases
FBS: Fasting blood sugar
HDL-c: High-density lipoprotein cholesterol
H-Tens: Hypertensive patients
HWE: Hardy–Weinberg equilibrium
K: Potassium
LDL-c: Low-density lipoprotein cholesterol
Na: Sodium
NO: Nitric oxide
OR: Odds ratio
PCR: Polymerase chain reaction
PCR-RFLP: Polymerase chain reaction-restriction fragment length polymorphism
SD: Standard deviation
SNPs: Single nucleotide polymorphisms
TC: Total cholesterol
TG: Triglycerides
